# Early real-time detection algorithm of tomato diseases and pests in the natural environment

**DOI:** 10.1186/s13007-021-00745-2

**Published:** 2021-04-23

**Authors:** Xuewei Wang, Jun Liu, Xiaoning Zhu

**Affiliations:** 1 Shandong Provincial University Laboratory for Protected Horticulture, Blockchain Laboratory of Agricultural Vegetables, Weifang University of Science and Technology, Weifang, 262700 Shandong China; 2 Elite Digital Intelligence Technology Co., LTD, Beijing, China

**Keywords:** Real-time detection algorithm, Deep learning, Dilated convolution, NMS, YOLOv3, Tomato diseases and pests, Natural environment

## Abstract

**Background:**

Research on early object detection methods of crop diseases and pests in the natural environment has been an important research direction in the fields of computer vision, complex image processing and machine learning. Because of the complexity of the early images of tomato diseases and pests in the natural environment, the traditional methods can not achieve real-time and accurate detection.

**Results:**

Aiming at the complex background of early period of tomato diseases and pests image objects in the natural environment, an improved object detection algorithm based on YOLOv3 for early real-time detection of tomato diseases and pests was proposed. Firstly, aiming at the complex background of tomato diseases and pests images under natural conditions, dilated convolution layer is used to replace convolution layer in backbone network to maintain high resolution and receptive field and improve the ability of small object detection. Secondly, in the detection network, according to the size of candidate box intersection ratio (IOU) and linear attenuation confidence score predicted by multiple grids, the obscured objects of tomato diseases and pests are retained, and the detection problem of mutual obscure objects of tomato diseases and pests is solved. Thirdly, to reduce the model volume and reduce the model parameters, the network is lightweight by using the idea of convolution factorization. Finally, by introducing a balance factor, the small object weight in the loss function is optimized. The test results of nine common tomato diseases and pests under six different background conditions are statistically analyzed. The proposed method has a F1 value of 94.77%, an AP value of 91.81%, a false detection rate of only 2.1%, and a detection time of only 55 Ms. The test results show that the method is suitable for early detection of tomato diseases and pests using large-scale video images collected by the agricultural Internet of Things.

**Conclusions:**

At present, most of the object detection of diseases and pests based on computer vision needs to be carried out in a specific environment (such as picking the leaves of diseases and pests and placing them in the environment with light supplement equipment, so as to achieve the best environment). For the images taken by the Internet of things monitoring camera in the field, due to various factors such as light intensity, weather change, etc., the images are very different, the existing methods cannot work reliably. The proposed method has been applied to the actual tomato production scenarios, showing good detection performance. The experimental results show that the method in this study improves the detection effect of small objects and leaves occlusion, and the recognition effect under different background conditions is better than the existing object detection algorithms. The results show that the method is feasible to detect tomato diseases and pests in the natural environment.

## Background

Agriculture is an important foundation for economic development. At present, agricultural output is largely limited by the disasters caused by plant growth disorders, which are characterized by great variety, large impact and easy transmission, and cause significant losses to agricultural production. Modern agricultural production methods are gradually moving closer to automation of unmanned machines, such as automatic irrigation technology in farmland [1], combined harvesting [2] and agricultural robots [3] and other equipment to improve the efficiency and output of production operations. With the development of science and technology and the improvement of life, artificial intelligence has gradually entered human life, and the application based on machine vision algorithm has been widely used in life, such as vehicle detection [4, 5], pedestrian detection [6, 7], safety production [8, 9] and fruit detection [10, 11] and other practical applications, They have replaced the traditional manual operation and greatly improved the production efficiency. Non-destructive detection and early identification of plant growth disorders are key to the development of precision agriculture and ecological agriculture. Early detection and prevention can effectively slow down the spread of plant growth disorders. Thus, it needs to adopt appropriate algorithm for accurate detection.

Among many agricultural products, tomato is the only vegetable that can be eaten as fruit, and its nutritional value is much higher than that of fruit [12]. Its yield is high, and its planting area is growing, especially in greenhouses where the planting area increases rapidly [13]. However, tomato is susceptible to diseases and pests during their growth, which seriously affects its yield and quality and causes enormous economic losses to farmers [14]. The outbreak is uncertain due to the diversity of tomato diseases and pests [15], the types of chemical sprays are diverse and the cost of treatment is often higher than that of prevention. According to the actual investigation of growers, as for the tomato planting base in Shouguang, Shandong Province, during the production period of one season tomato, up to 5 kinds of agricultural chemicals are sprayed, the number of times of spraying was up to 10, and the chemical pesticide used for diseases and pests control was up to 1000 tons [16]. Pesticide abuse not only destroys the ecological balance of farmland, but also increases the resistance of pests and the cost of control, resulting in serious negative effects of pesticide control [17, 18]. Therefore, if the real-time diseases and pests detection and early recognition and early warning can be carried out for tomatoes, the occurrence of tomato plant growth disorders can be identified timely and accurately, and the producers can be guided accurately to carry out plant protection and control, and the plant growth disorders can be controlled in the early stage of occurrence, the production goal of improving tomato yield and quality can be achieved, and the pesticide spray can be reduced. Consequently, it is necessary to study the characteristics of tomato diseases and pests, and to recognize and judge the disasters as early as possible.

Traditional identification and early warning judgment of tomato diseases and pests are mainly based on field survey by plant protection experts and experienced tomato producers according to the growth status of tomato [19]. Only relying on human resources to identify the information of plant growth disorders is labour-intensive, time-consuming and slow, and some deviations may occur, thus hindering the timely treatment of diseases and pests [20]. Therefore, today’s agricultural production urgently needs a new system to liberate producers from inefficient, complex diseases and pests identification processes.

In recent years, due to the rapid development of deep learning related theories and the improvement of computational ability, deep convolutional networks have achieved great success in computer vision. In terms of object detection, the accuracy of deep learning-based methods greatly exceeds that of traditional methods based on manual design features such as HOG and SIFT [21]. Object detection is to draw a range of objects of interest in an image, then select the target box with a rectangle and label it with a category. Deep learning-based object detection mainly includes two types, one is convolutional network structure based on region generation, and the representative network are R-CNN [22], faster R-CNN [23]; the other is to treat the detection of the object location as a regression problem, directly using the CNN network structure to process the entire image, and at the same time to predict the category and location of the object, the representative networks are: SSD [24], YOLO [25], YOLO9000 [26], YOLOv3 [27].

At present, most studies focus on image classification of crop diseases and pests based on deep learning. There are few studies on object detection of crop diseases and pests based on deep learning [28–31]. The existing object detection methods of tomato diseases and pests based on deep learning mostly use the algorithm based on region suggestion for detection [28, 32–35], the accuracy has greatly improved compared with traditional methods, but the object detection process takes a long time and it is difficult to detect and locate tomato diseases and pests in real-time under natural conditions.

It is now possible to collect images of diseases and pests in real-time using the Internet of Things and video camera equipment in tomato greenhouses, then transmit them to remote computers through the Internet, and then use computers to automatically identify the types of diseases and pests. However, the recognition result is closely related to the image quality acquired. Disease acquisition is affected by the quality of the camera, light, shooting level, etc. If the lesion is too small, the image is blurred, there are spots, shadows, etc., it will seriously affect the correct recognition. Due to the complex diversity of tomato diseases and pests images under real natural conditions, especially the image of diseases and pests collected by Internet of Things video equipment is massive, highly redundant and noisy, the feature extraction ability of existing methods is not suitable for tomato diseases and pests detection under natural conditions. Previous studies have shown high accuracy under controlled laboratory conditions, but in complex light and complex background, the object detection results are not ideal and face many challenges [36]. Our previous work has achieved good results on the detection of a common gray leaf spot disease of tomato under natural conditions [37]. Chen et al. [38] collected 8616 images containing five kinds of tomato diseases on the spot. The images were denoised and enhanced by combining the binary wavelet transform of Retinex (BWTR). The two-channel residual attention network model (B-ARNet) was used to identify the images with an accuracy of about 89%. Pattnaik et al. [39] proposed a pre-trained deep CNN framework for transfer learning for pest classification in tomato plants, and achieved the highest classification accuracy of 88.83% using DenseNet 169 model. However, in actual production, tomato may suffer from a variety of diseases or pests at the same time.

To solve the above problems, this study takes 9 common diseases and pests of tomato as the research object, uses machine vision object detection method for diseases and pests detection, and based on the deep learning YOLOv3 object detection algorithm, through the improvement of YOLOv3 algorithm to obtain a better detection network model, which achieves the early detection of tomato diseases and pests under natural conditions. Firstly, in view of the complex background of tomato diseases and pests images under natural conditions, a dilated convolution layer [40] was used to replace the convolution layer in the backbone network, so that it can maintain high resolution and receptive field, and improve the ability of small size object detection. Secondly, in the detection network, the non-maximum suppression (NMS) algorithm [41] is used according to the size of the intersection union ratio ($IoU$) of candidate boxes predicted by multiple grids and the linear attenuation confidence score, remove the predictive boxes with larger $IoU$ and lower confidence scores, the prediction box with higher confidence score is retained as the object detection box. The obscured tomato diseases and pests objects are retained to solve the detection problem of mutual obscure tomato diseases and pests objects. Thirdly, to reduce the model volume and reduce the model parameters, the network is lightweight by using the idea of convolution factorization. Finally, aiming at the missed detection of small size tomato diseases and pests objects in the detection process, a loss function improvement method based on balance factor is proposed to balance the difficulty of samples to obtain better results.

## Materials and methods

### Dataset used in the research

**Fig. 1:**
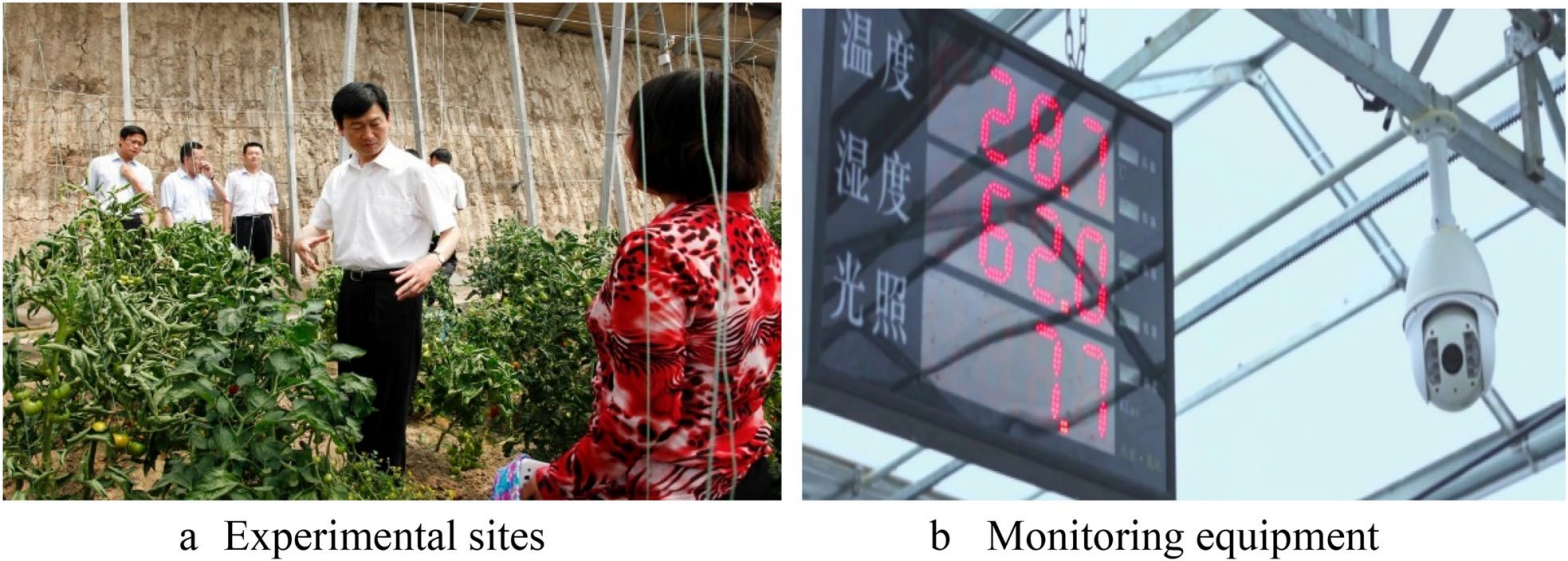
Video monitoring system of agricultural internet of things

To verify the validity of the object detection method for tomato diseases and pests proposed in this study and to ensure timely detection in the early stage of diseases and pests occurrence, the growth status of tomato was monitored in real-time by using the video monitoring system in the tomato greenhouse in Shouguang City, Shandong Province. The greenhouse dimensions are 8 m north–south span and 80 m east–west length. The video monitoring system is shown in Fig. 1 (The website of the system is: http://139.224.3.180/farming/loginController.do?login).

#### Hardware environment

The video monitoring system consists of three network cameras, two switches, two wireless sending and receiving network bridges, one network video recorder, one streaming server and one wireless router, one central management server (containing intelligent analysis module), one video storage server, several network lines, and one mobile phone for testing.

#### Software environment

Real-time video monitoring module. This module is primarily designed to provide users with live video information, where users can view real-time live video and historical video data. Video acquisition module. This module is the core module of the system, which provides real-time intelligent acquisition of monitoring video data.

The video monitoring camera can perform 23× optical zooms. The growth status of single leaf and single fruit can be observed within 500 m, which is convenient for the manager to observe tomato growth remotely. To reduce the workload of video acquisition, we try to collect in high attack period of disease, take videos at multiple angles, and the data can be queried and downloaded at any time.

From March to May of 2019 and 2020, tomato diseases and pests images were collected by surveillance video. The resolution was 1960 × 1080 pixels. The focus was on tomato leaf diseases and pests images. The monitoring includes tomato growth status in different periods, different locations and different weather. At each video monitoring point, when the computer judges that there were suspected lesions in the tomato growth process, the videos were detected and tracked every day, then the images of the best leaf postures were captured and saved in JPEG format. To determine the best leaf postures, the leaves with the gross good postures were manually identified first, the dimensions of the bounding box of this class of leaves were manually scaled, and K-means clustering was performed on the aspect ratio of the bounding box, with the cluster centre as the optimal length and width of the tomato leaf. Cluster centre was found to be 1: 0.79 by experiment, so we finally determined the best leaf postures when the length to width ratio of the detected leaf bounding box was equal to or close to 1: 0.79. Compared with the traditional manual photography data acquisition method, this acquisition method is easy to collect tomato diseases and pests images rich in complex natural environment information, including various background interference information such as leaves, weeds and soil, which is suitable for the mobile end extended application and can be deployed in tomato greenhouses.

In most of the images initially collected, the object subject to be studied, i.e. the lesion part in the image, only accounts for a small part of the whole image. To reduce the amount of data in post-processing, improve the processing efficiency and eliminate the interference caused by non-subject parts as far as possible, the redundant parts were removed by image clipping only retaining the main part of the study. In image clipping, the tool used was Adobe Photoshop CS6 × 64. To make the size of all kinds of tomato diseases and pests images in the dataset consistent, a function cv2.resize() in Open CV was called through normalization operation, and the size of the images was unified to 256 × 256 size. Because the length–width ratios of the disease images are close, actual shapes of lesions or pests will not get altered after the uniform size.

Similar sample images were filtered out by manual screening, and some invalid data was removed. The early images of 7 common diseases and 2 pests of tomato were selected, and a total of 10,696 images were captured. The images are randomly divided into training dataset, validation dataset and test dataset according to the proportion of 70%, 20% and 10%. The early detection experiments of diseases and pests can be carried out.

The images containing the characteristics of tomato diseases and pests were annotated by labelImg. The labelImg software is an image annotation tool for deep learning dataset based on Python language, which is mainly used to record the category name and position information of the object, and store the information in the Extensible Markup Language (XML) format file.

The specific annotation process is as follows: Firstly, tomato diseases and pests characteristics in the image are identified manually to determine that they are tomato diseases and pests objects; then, tomato diseases and pests objects are selected sequentially with a vertical minimum external rectangular box, and category labels are set at the same time. The label information (x, y, h, w) of the rectangular box is stored in the label XML file, where (x, y) is the upper left coordinate of the rectangular box and (h, w) is the height and width of the rectangular box, respectively. According to YOLOv3 training requirements, the XML file is transformed into a text format (TXT) file with the content of object category and coordinates, which is prepared for the next training.

The dataset constructed in this study contains 10,696 pictures. The total number of tomato diseases and pests objects under different background conditions is 63,089, as shown in Table 1.

**Table 1:** Sample number of tomato diseases and pests

Serial number	Class	Number of images	Number of annotated samples (bounding boxes)
1	Early blight	1208	6127
2	Late blight	1101	7172
3	Powdery mildew	1227	5312
4	Spot blight	1112	6427
5	Gray mold	1227	7015
6	Leaf mold	1271	7121
7	Gray leaf spot	1177	7188
8	Leaf miner	1222	7559
9	Whitefly	1151	9168
Total		10,696	63,089

**Fig. 2:**
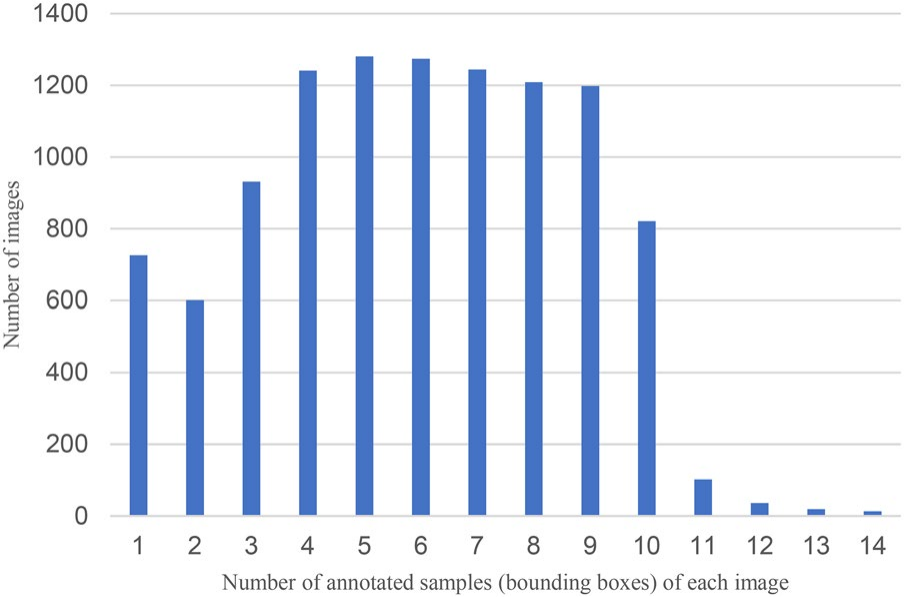
The number of tomato diseases and pests objects in each image

The statistical histogram of the number of tomato diseases and pests objects in each image in the dataset is shown in Fig. 2. The abscissa represents the number of annotated samples (bounding boxes) of each image, and the ordinate represents the number of images.

According to Fig. 2, the number of objects in each image in the dataset varies greatly, with 1 to 9 objects in most images and more than 10 objects in a few images. Considering that the early symptoms of tomato diseases and pests are not obvious and the size of disease spots is small, the object size of diseases and pests is statistically analysed, and it is found that the average pixel size of the object is 21.0967, and the ratio of the average pixel of the object to the original image is 0.0219. It can be seen that the object pixel size of tomato diseases and pests image is diverse and occupies a small proportion in the image. Therefore, the early objects of tomato diseases and pests are small objects, and there are a lot of background information in the images.

From the dataset constructed in this study, it can be concluded that the number of tomato diseases and pests objects varies greatly in different images. Tomato diseases and pest objects in the same image may have multiple scales, multiple locations, orientations and other distributions at the same time. Under different resolutions, the size and detailed texture of tomato diseases and pests objects will also be different. In terms of image size, tomato diseases and pests belong to small objects. Combined with the analysis of statistical histogram, it is known that the detection of tomato diseases and pests objects requires the study of a multi-scale detection network model, retaining detailed information, making full use of the spatial location information of tomato diseases and pests objects, etc. In conclusion, the dataset constructed in this study can be applied to the study of object detection algorithms for tomato diseases and pests. When constructing the object detection model, it is necessary to focus on the detailed information and multi-scale features of tomato diseases and pests objects.

### Principle of the improved YOLOv3 model

**Fig. 3:**
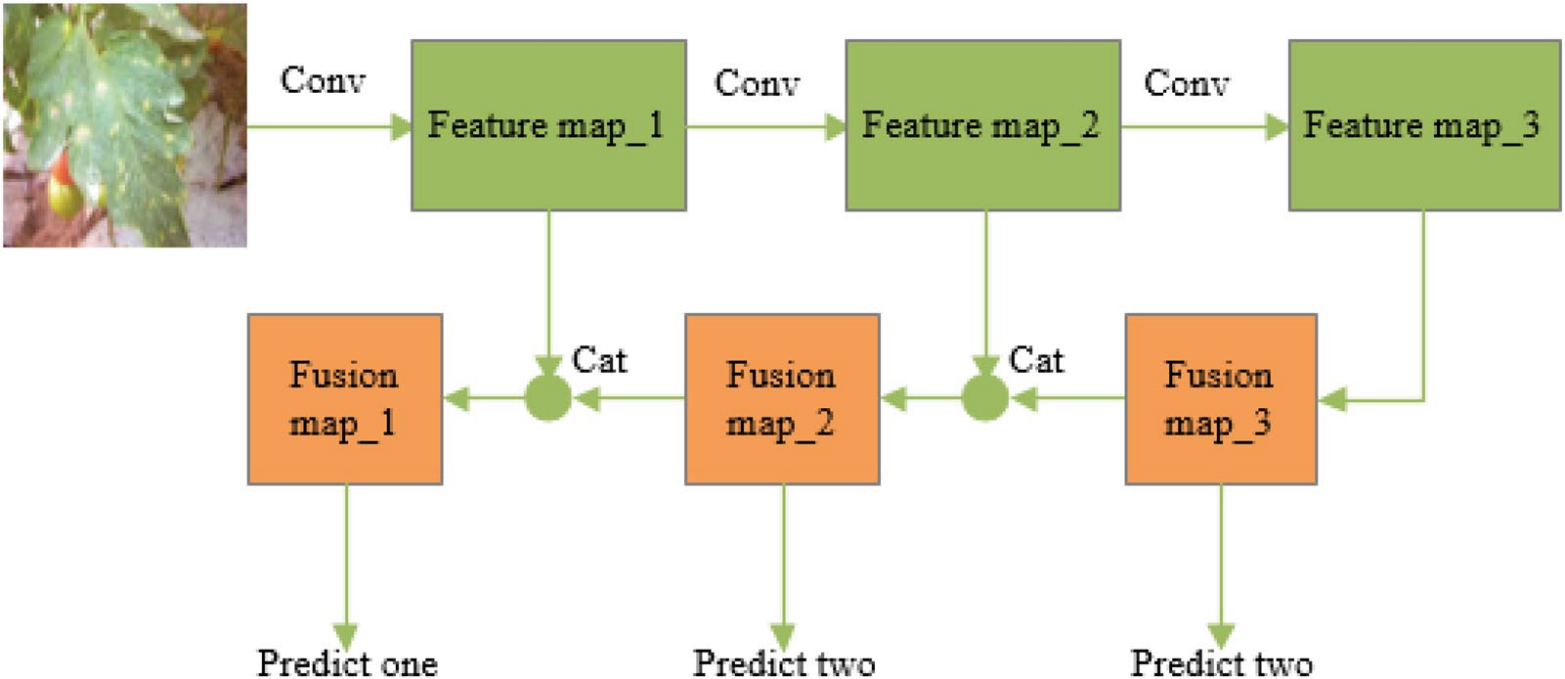
The main flow of the algorithm

The features of tomato diseases and pest objects in the image are extracted using deep convolution neural network, and the detection and localization of the objects are achieved by regression. The main flow of the algorithm is shown in Fig. 3. Firstly, feature extraction network is constructed by residual module to obtain image object feature pyramids; then, features of different depths are fused by feature fusion mechanism, and the location information of the object is predicted by regression on the fused feature map, and the confidence score is predicted by Sigmiod function; finally, the output is filtered by NMS (non-maximum suppression).

#### Problems of YOLOv3 model

YOLO series object detection network is the most representative network structure in one stage object detection network. YOLOv3 is the latest improved network of YOLO series because the detection accuracy can be comparable to two target detection networks and can achieve real-time detection speed, so it has become one of the most popular object detection algorithms. Considering that the object detection of tomato diseases and pests needs to take into account both the accuracy and speed of detection in practical application, this study takes YOLOv3 as the main body and improves the algorithm according to the application scenario of tomato diseases and pests object detection to complete the location and class identification of tomato diseases and pests.

YOLOv3 network, which has been improved many times, has achieved a good balance between detection accuracy and detection speed and has become the preferred algorithm for many object detection tasks because of its simple implementation. However, as a single object detection network, there are still problems of large positioning error, unbalanced prospects and background complexity.

To pursue detection speed, YOLOv3 algorithm integrates object location and classification into a convolutional neural network, and simultaneously predicts the location coordinates and class information of the object. However, because the deep feature map in the convolutional neural network contains more advanced and abstract feature information, which is suitable for object classification, but because of the loss of more spatial information, it has poor effect on object localization. Shallow feature maps are more specific and contain more spatial information, which is suitable for coordinate positioning but not ideal for object classification. Although YOLOv3 tries to use the concatenation of deep feature map and shallow feature map to fuse different levels of feature information, there is still the problem of inaccurate object location compared with two-stage object detection algorithms.

#### The improved backbone network

YOLOv3 uses Darknet-53 as a feature extraction network and achieves good object detection results on common datasets. Compared with the objects in common datasets, tomato diseases and pests have smaller objects, and multi-object aggregation often occurs. The background in the natural environment is complex and seriously affected by light conditions. It is difficult to extract significant features from images.

The residual network used by Darknet-53 solves the problem of gradient disappearance during propagation. However, each residual unit contains only two convolution layers, which limits the capacity of the unit to some extent. Simply increasing the width or depth of each unit saturates the network performance [42]. According to Szegedy et al. [43], the improvement of network performance is related to the diversity of network structure, not only increasing the depth or width of the network. In this study, from the perspective of structural diversity, the backbone network was redesigned according to the characteristics of tomato diseases and pests objects. For the detection of small objects, on the one hand, high-resolution feature maps are needed to detect the object information in a small area; on the other hand, a wider receptive field or more global information is needed to accurately determine the location and semantic features of the objects. To improve the detection of small objects in tomato diseases and pests images, a backbone network with high resolution and large receptive fields is proposed for feature extraction by combining dilated convolution and FPN (feature pyramid) structure.

**Fig. 4:**
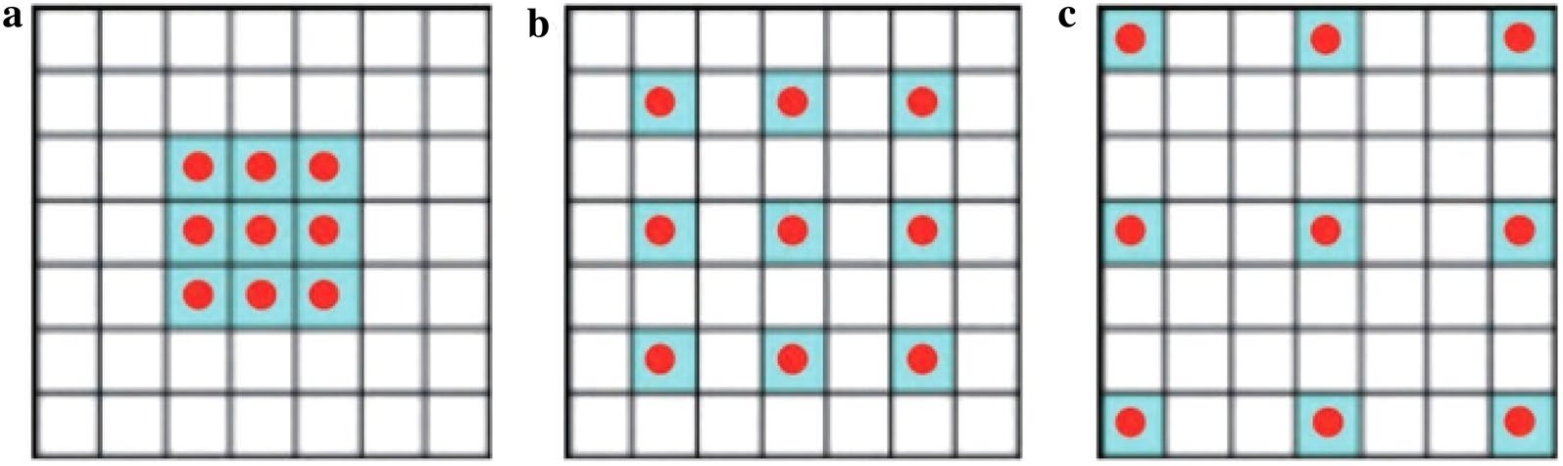
Dilated convolution kernels with different intervals **a** ${r}_{rate}=1$; **b** ${r}_{rate}=2$; **c** ${r}_{rate}=3$

Dilated convolution enlarges the receptive field of convolution kernel by changing the internal spacing of convolution kernel. Figure 4 shows three kinds of dilated convolution kernels with different intervals. ${r}_{rate}$ represents the interval in the convolution kernels. Figure 4a represents the receptive filed of the convolution kernel is $3\times 3$ and ${r}_{rate}=1$. Figure 4b represents the receptive filed of the convolution kernel is $7\times 7$ and ${r}_{rate}=2$. Figure 4c represents the receptive filed of the convolution kernel is $15\times 15$ and ${r}_{rate}=3$. In this way, it can ensure that the convolutional neural network can extract the feature information in a larger receptive field.

**Fig. 5:**
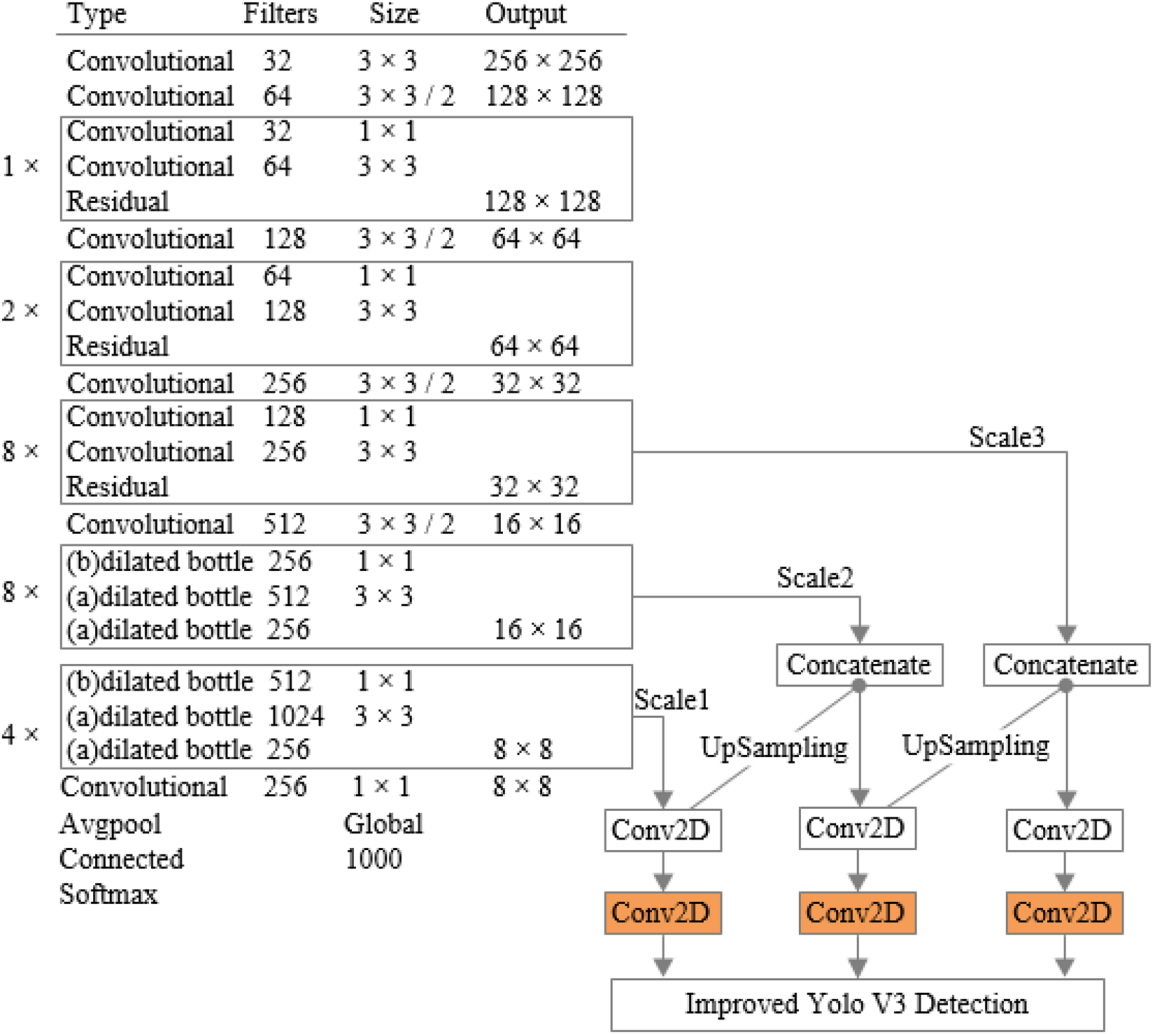
Backbone network of the improved YOLOv3

The backbone network of the improved YOLOv3 is shown in Fig. 5. The resolution of features will directly affect the detection of small objects and the overall performance indicators. Low resolution will lead to the loss of semantic features of small objects seriously, and high-resolution features will cause a large amount of computation and memory storage. Therefore, considering that the overall performance of backbone networks will not be reduced, compared with the original YOLOv3 backbone network, the improved YOLOv3 uses FPN structure to reduce the loss of semantic features of the small-scale object in deep network, which is conducive to identifying smaller objects.

**Fig. 6:**
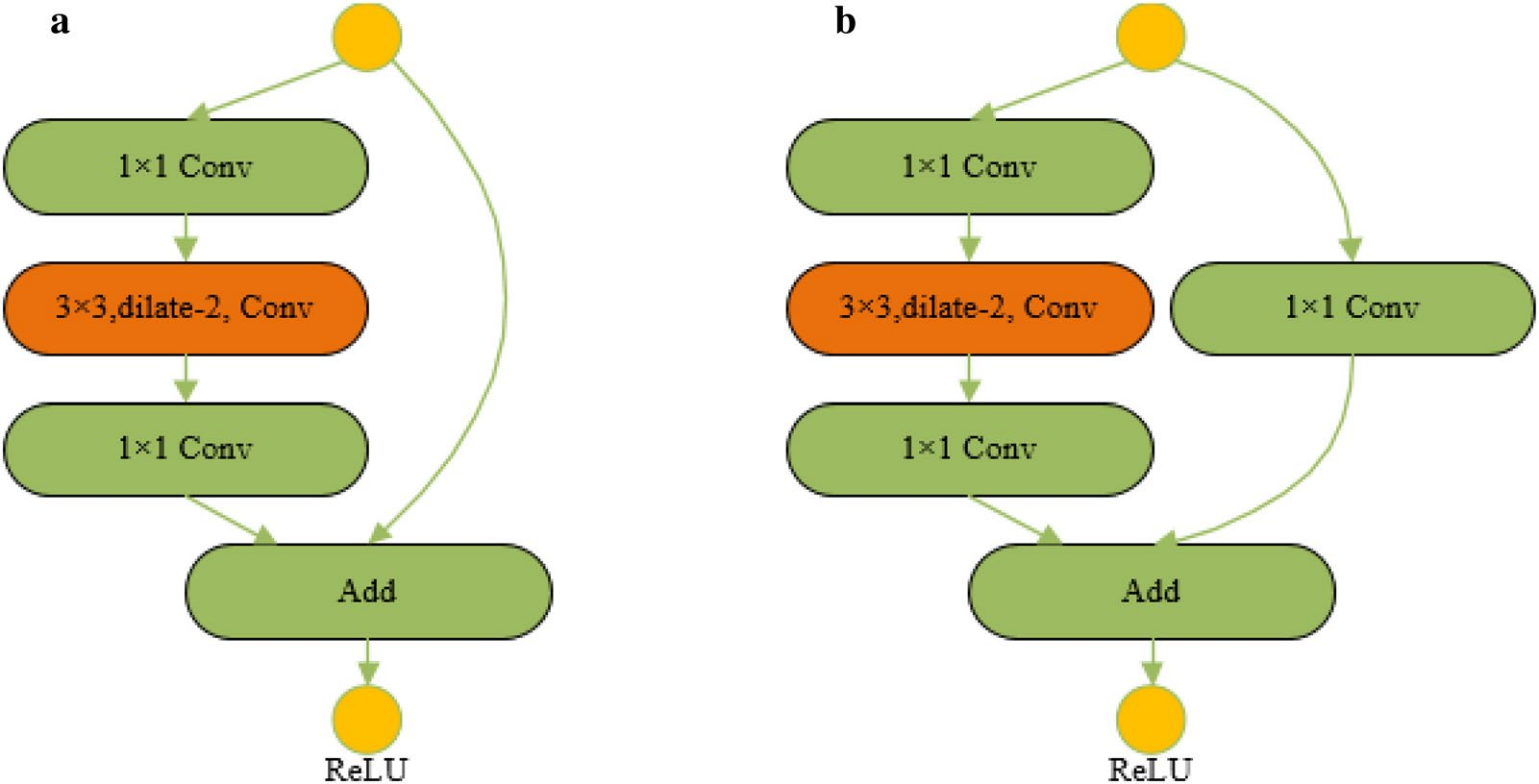
Structure of dilated convolution residuals. **a** Dilated convolution bottleneck; **b** dilated convolution bottleneck with $1\times 1$ Conv projection

The dilated convolution bottleneck layer is introduced as shown in Fig. 6a. Dilated convolution bottleneck with $1\times 1$ Conv projection is shown in Fig. 6b. In these two kinds of dilated convolution residual structures with lower complexity, Conv is convolutional layer; Add is addition operation, ReLU is the activation function. The receptive filed of the convolution kernel of the dilated convolution is $3\times 3$ and ${r}_{rate}=2$. Therefore, the receptive field and feature expression ability of the backbone network are increased as a whole. Meanwhile, the dilated convolution residual structure still has the advantages of fewer network parameters of residual units and lower computational complexity. Figure 6b uses $1\times 1$ Conv to achieve cross-channel feature fusion, which integrates feature information well.

Finally, the improved YOLOv3 can maintain a higher resolution and a larger receptive field of the feature map in the deep convolutional neural network, enhancing the receptive field and detection ability of the YOLOv3 algorithm for small objects.

#### Optimized linear attenuation NMS

The occlusion problem is one of the main factors that restrict the improvement of detection accuracy. Because of the close distance between the objects to be detected, missed detection or false detection occurs easily. During the test, the detection algorithm generates a set of candidate boxes around each suspected object. If the occlusion phenomenon is absent, NMS is performed on this set of candidate boxes. Redundant candidate boxes can be effectively filtered out, resulting in the final highest-scoring predicted box. However, when two or more objects occlude each other, it results in the final set of several candidate boxes being fused into one group, at which point the algorithm cannot tell whether the candidate box comes from the same object or several different objects, which will lead to missed detection or false detection. To improve the precision of detection in the context of occlusion, we should try to make candidate boxes generated by different objects distinguishable and screen them individually.

**Fig. 7:**
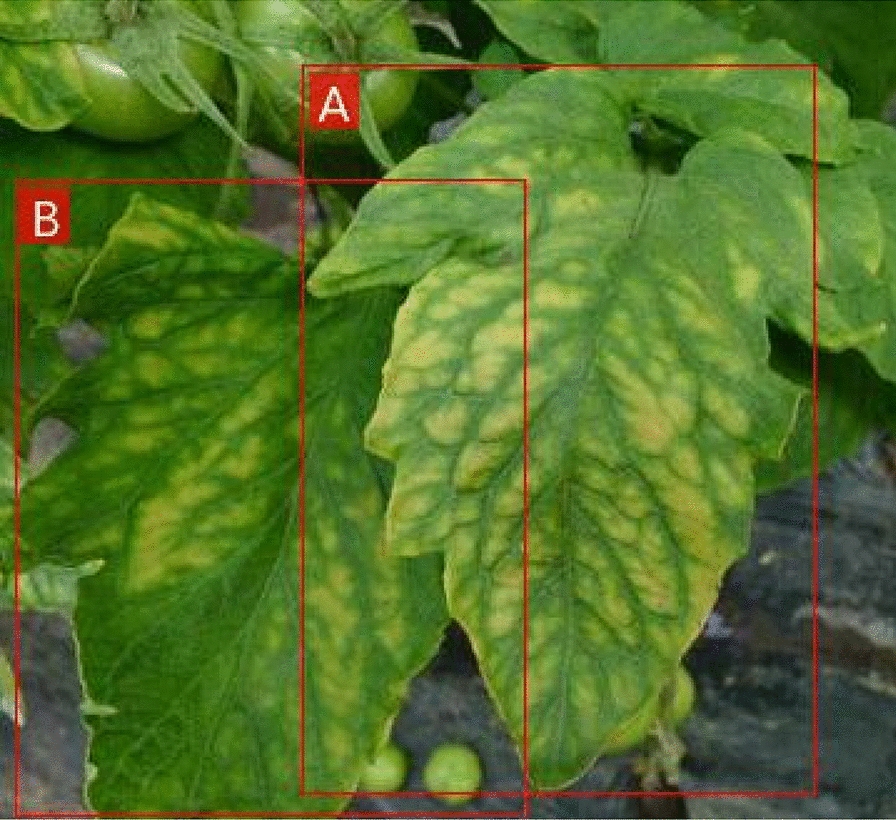
Two tomato leaves of occlusion

As shown in Fig. 7a, b are two images from tomato diseases and pests dataset. The confidence score of the A-leaf prediction box is 0.8, the B-leaf prediction box is 0.6, and the A-leaf obscures the B-leaf. The intersection over the union of the prediction boxes of A and B leaves is $IoU>0.5$. The NMS algorithm is used to process redundant prediction boxes. When $IoU>0.5$, because the set threshold of YOLOv3 is 0.5, the A-leaf with higher confidence score is retained, and the confidence score of the prediction box of B-leaf is set to 0, thus the result of B-leaf cannot be detected.

In this study, a linear attenuation NMS algorithm is used to solve the problem that the occluded leaves could not be accurately detected due to NMS in the original YOLOv3. When the $IoU$ is higher than the suppression threshold, the confidence score in the NMS is linearly smoothed. The optimized NMS algorithm is expressed as

1 $$S_{confi}^{*} = \left\{ {\begin{array}{*{20}l} {S_{confi} , IoU\left( {M,b_{i} } \right) \le N_{t} } \\ {S_{confi} \left[ {1 - IoU\left( {M,b_{i} } \right)} \right] , IoU\left( {M,b_{i} } \right) > N_{t} } \\ \end{array} } \right.$$

In the above-mentioned formula, ${S}_{confi}^{*}$ is the confidence score after linear smoothing treatment, ${S}_{confi}$ is the confidence score of the original NMS, $M$ is the prediction box with the higher confidence score, ${b}_{i}$ is the object prediction box to be compared, $IoU\left(M,{b}_{i}\right)$ is the intersection over union of $M$ and ${b}_{i}$,${N}_{t}$ is the suppression threshhold.

**Fig. 8:**
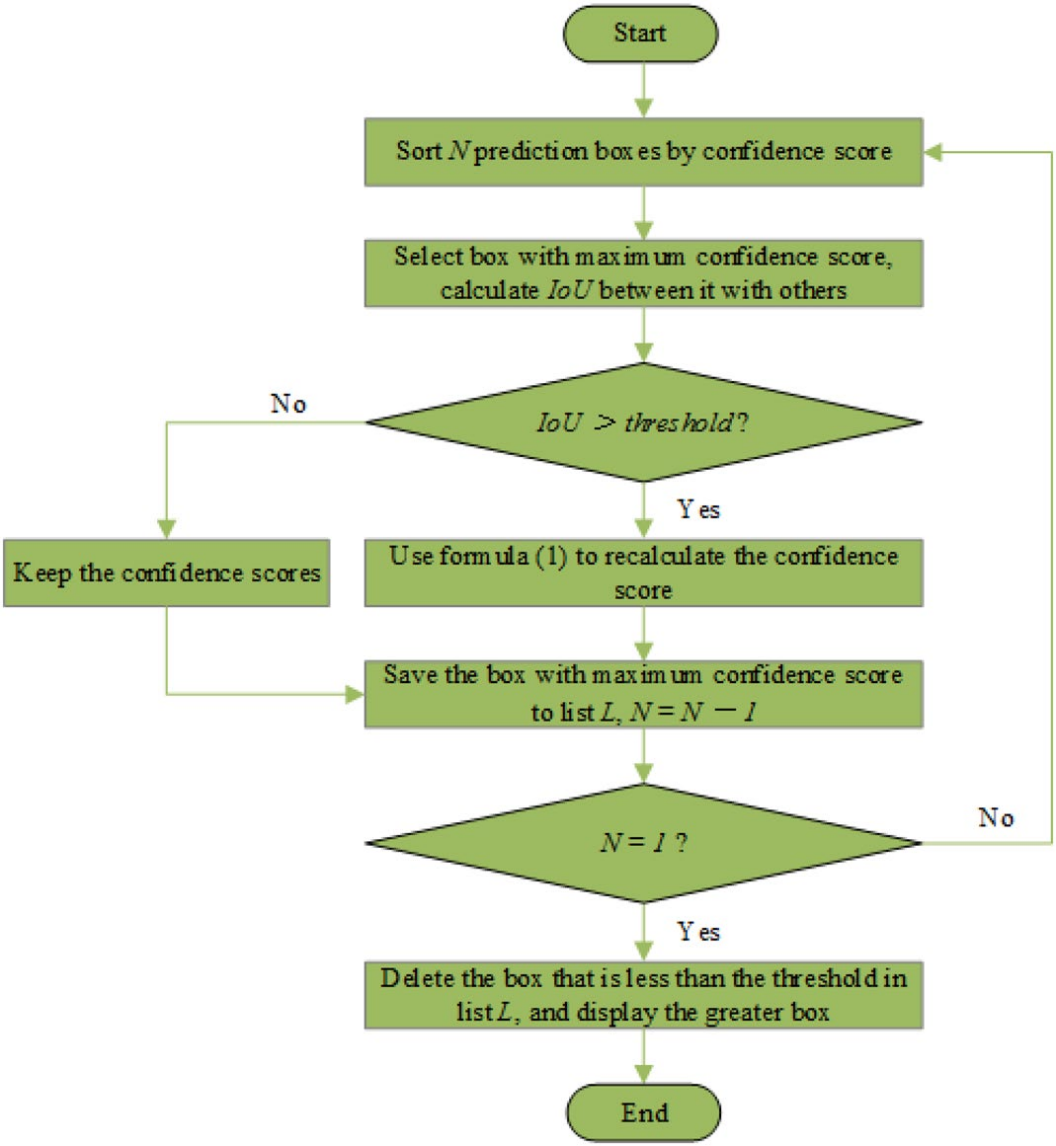
Flow chart of the optimized NMS processing

The flow chart of the optimized NMS algorithm is shown in Fig. 8.

The specific steps are as follows:

1. According to the size of the confidence score, the $N$ prediction boxes generated by regression are sequentially sorted;
2. Select the prediction box with the largest confidence score and calculate the $IoU$ values with other prediction boxes;
3. Compare the calculated $IoU$ value with the threshold value, if the $IoU$ value is greater than the threshold value, the confidence score will be recalculated using formula (1). Otherwise the original value will remain unchanged;
4. Store the prediction box with the largest confidence score in $L$, execute $N-1$, and repeat steps 1)–4) for the remaining prediction box until $N=1$, that is, all prediction boxes are processed;
5. Delete the prediction box with the confidence score less than the threshold in $L$, and display the prediction box with the confidence score greater than the threshold; that is, the prediction box is the final detection result.

The improved NMS algorithm is no longer a simple threshold comparison method, but a method by using the linear attenuation confidence score of the $IoU$ value, which can avoid the false deletion of the prediction box of the occluded object and improve the detection ability of the occluded object.

#### Lightweight processing of the model

**Fig. 9:**
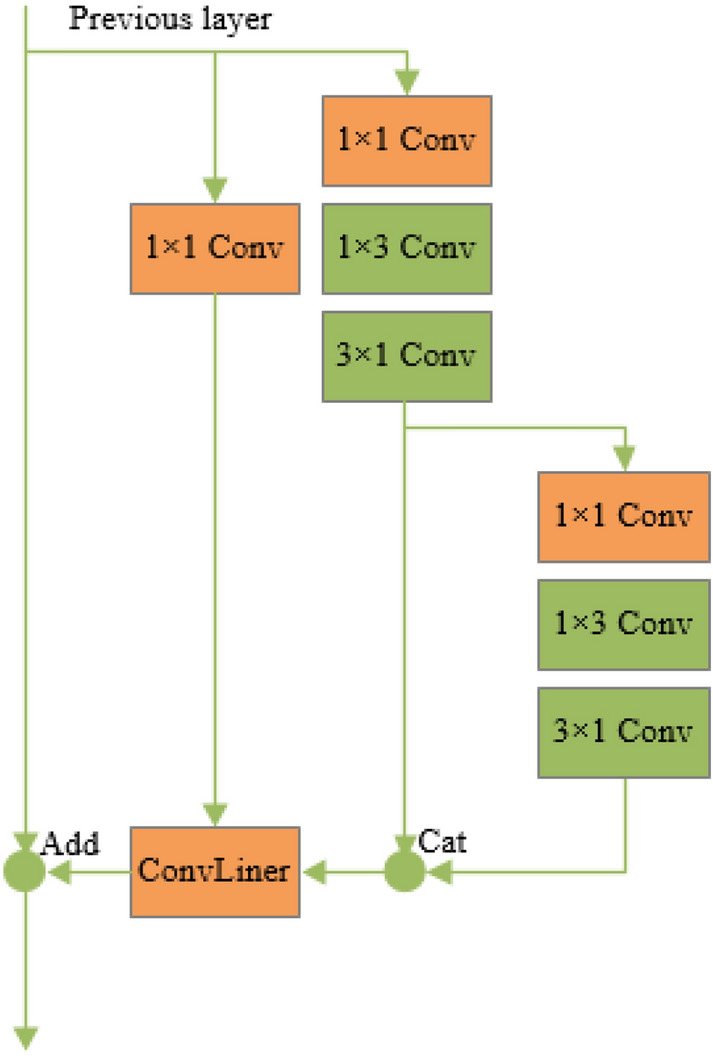
Convolution factorization

The improved network increases the diversity of structures and the parameters in the network become more numerous. When the dataset is small, too many network parameters will lead to over-fitting problems, which can not make the model have good generalization ability but also increase the computational difficulty and make the network difficult to train. To solve the above problems, the model is lightweight processed in this study. The idea of convolution factorization is introduced into the network, and the larger two-dimensional convolution is decomposed into the smaller one-dimensional asymmetric convolution, as shown in Fig. 9. On the one hand, the model parameters can be reduced, the network operation can be accelerated, and the overfitting of the model can be avoided; on the other hand, the non-linear layer of the model can be expanded by the superposition of asymmetric convolutions, which can obtain more and richer spatial features and increase the diversity of features.

To compare the number of parameters before and after the network improvement, the number of parameters of the network is calculated by formula (2):

2 $$S = \mathop \sum \limits_{l = 1}^{D} K_{l}^{2} \cdot C_{l - 1} \cdot C_{l}$$

In the above-mentioned formula, $S$ is the number of network parameters, $K$ is the size of convolution kernel, $C$ is the number of convolution channels, $D$ is the number of network layers. The calculation results are shown in Table 2.

**Table 2:** Comparison on parameter numbers

Network model	Parameter numbers
Original network model	$4.05\times {10}^{8}$
The new network model	$7.92\times {10}^{8}$
The new network model with convolution factorization	$4.36\times {10}^{8}$

It can be seen that the number of the improved network parameters has increased. However, by introducing the convolution factorization idea, the number of parameters is effectively reduced, and the increase of parameters is significantly reduced.

#### The improved loss function

The two sub-tasks of object detection are bounding box prediction and category prediction. To accomplish these two sub-tasks, the original YOLOv3 algorithm’s loss function design includes three parts, namely coordinate prediction, confidence prediction and category prediction.

The loss function of the object detection network of YOLOv3 is shown in Formula (3).

3 $$Loss = Loss_{coord} + Loss_{obj} + Loss_{class}$$

In the above formula, $Los{s}_{coord}$ is coordinate prediction loss, $Los{s}_{obj}$ is confidence prediction loss and $Los{s}_{class}$ is category prediction loss.

4 $$\begin{aligned} Loss_{coord} & = \lambda_{coord} \mathop \sum \limits_{i = 0}^{{S^{2} }} \mathop \sum \limits_{j = 0}^{B} l_{ij}^{obj} \left[ {(x_{i} - \hat{x}_{i} )^{2} + (y_{i} - \hat{y}_{i} )^{2} } \right] \\ & \quad + \lambda_{coord} \mathop \sum \limits_{i = 0}^{{S^{2} }} \mathop \sum \limits_{j = 0}^{B} l_{ij}^{obj} \left[ {(\sqrt {w_{i} } - \sqrt {\hat{w}_{i} } )^{2} + (\sqrt {h_{i} } - \sqrt {\hat{h}_{i} } )^{2} } \right] \\ \end{aligned}$$

In the above formula, ${\lambda }_{coord}$ is weight coefficient, ${S}^{2}$ is the number of grids of the input image, $B$ is the number of bounding boxes predicted by a single grid, ${l}_{ij}^{obj}$ represents when the grid *i* predict the bounding box *j* and an object is detected, the value is set to 1. Otherwise 0, ${x}_{i}$ is the abscissa of the centre point of the predicted bounding box, ${y}_{i}$ is the ordinate of the centre point of the predicted bounding box, ${\widehat{x}}_{i}$ is the abscissa of the centre point of the actual bounding box, ${\widehat{y}}_{i}$ is the ordinate of the centre point of the actual bounding box,${w}_{i}$ is the width of the predicted bounding box, ${h}_{i}$ is the height of the predicted bounding box, ${\widehat{w}}_{i}$ is the width of the actual bounding box, ${\widehat{h}}_{i}$ is the height of the actual bounding box.

5 $$Loss_{obj} = \mathop \sum \limits_{i = 0}^{{S^{2} }} \mathop \sum \limits_{j = 0}^{B} l_{ij}^{obj} (C_{i} - \hat{C}_{i} )^{2} + \lambda_{noobj} \mathop \sum \limits_{i = 0}^{{S^{2} }} \mathop \sum \limits_{j = 0}^{B} l_{ij}^{obj} (C_{i} - \hat{C}_{i} )^{2}$$

In the above formula, ${C}_{i}$ is the predicted confidence score of the object, $\widehat{C}$ is the actual confidence score of the object.

6 $$Loss_{class} = \mathop \sum \limits_{i = 0}^{{S^{2} }} l_{ij}^{obj} \mathop \sum \limits_{c \in class} \left( {p_{i} \left( c \right) - \hat{p}_{i} \left( c \right)} \right)^{2}$$

In the above formula, $c$ is the category of the detected object, ${p}_{i}(c)$ is when the *i* grid detects an object, the prediction probability of the object belonging to the category $c$, ${\widehat{p}}_{i}\left(c\right)$ is when the *i* grid detects an object, the actual probability of the object belonging to the category $c$.

The loss function of coordinate prediction and confidence prediction is to ensure the accuracy of bounding box regression. After the adaptive dimension clustering of anchor bounding box, the accuracy of bounding box regression has been improved correspondingly. Another sub task, the accuracy of category prediction becomes more important.

It is found that the average accuracies of tomato diseases and pests objects are different. Because of the variety of pest forms and the small size of the early objects, there will be many kinds of attitudes when they gather and overlap each other. Therefore, unlike diseases, it is difficult to learn the characteristics of pests, and it is easy to miss the detection of small-scale objects. At the same time, different classes of diseases and pests have different lesion sizes. To narrow the gap between them, this study adds a balance factor to each category and weighs the difficulty of samples among different categories. The modified loss function of category prediction is as follows:

7 $$Loss_{class}^{^{\prime}} = \mathop \sum \limits_{i = 0}^{{S^{2} }} l_{ij}^{obj} \alpha_{c \in class} \mathop \sum \limits_{c \in class} (p_{i} \left( c \right) - \hat{p}_{i} \left( c \right))^{2}$$

In the above formulas, by adjusting the balance factor ${\alpha }_{c\in class}$, it makes the model find the best point between the bounding box prediction and category prediction. It makes the algorithm get the best detection effect.

The final improved loss function is as follows:

8 $$\begin{aligned} Loss^{^{\prime}} & = Loss_{coord} + Loss_{obj} + { }Loss_{class}^{^{\prime}} \\ & = \lambda_{coord} \mathop \sum \limits_{i = 0}^{{S^{2} }} \mathop \sum \limits_{j = 0}^{B} l_{ij}^{obj} \left[ {(x_{i} - \hat{x}_{i} )^{2} + (y_{i} - \hat{y}_{i} )^{2} } \right] + \lambda_{coord} \mathop \sum \limits_{i = 0}^{{S^{2} }} \mathop \sum \limits_{j = 0}^{B} l_{ij}^{obj} \left[ {(\sqrt {w_{i} } - \sqrt {\hat{w}_{i} } )^{2} + (\sqrt {h_{i} } - \sqrt {\hat{h}_{i} } )^{2} } \right] \\ & \quad + \mathop \sum \limits_{i = 0}^{{S^{2} }} \mathop \sum \limits_{j = 0}^{B} l_{ij}^{obj} (C_{i} - \hat{C}_{i} )^{2} + \lambda_{noobj} \mathop \sum \limits_{i = 0}^{{S^{2} }} \mathop \sum \limits_{j = 0}^{B} l_{ij}^{obj} (C_{i} - \hat{C}_{i} )^{2} + \mathop \sum \limits_{i = 0}^{{S^{2} }} l_{ij}^{obj} \alpha_{c \in class} \mathop \sum \limits_{c \in class} (p_{i} \left( c \right) - \hat{p}_{i} \left( c \right))^{2} \\ \end{aligned}$$

### Metrics used to evaluate the proposed method

In this study, F1 score and AP (average precision) evaluate the model trained by the loss function. The formula is expressed as follows.

9 $$P = \frac{TP}{{TP + FP}}$$

10 $$R = \frac{TP}{{TP + FN}}$$

11 $$F1 = \frac{2PR}{{P + R}}$$

12 $$AP = \mathop \smallint \limits_{0}^{1} P\left( R \right)dR$$

In the above-mentioned formula, P is the accuracy rate, R is the recall rate. TP is the number of true positive samples. FP is the number of false-positive samples. FN is the number of false-negative samples.

### Experimental operation environment

The experimental hardware environment of this study is shown in Table 3. On this basis, the software environment is built as follows: Ubuntu 16.04, python, OpenCV and CUDA. The framework uses Caffe and Darknet-53 frameworks.

**Table 3:** Configuration of experimental hardware environment

Hardware name	Model	Number
Main board	Asus WS X299 SAGE	1
CPU	INTEL I7-9800X	1
Memory	The Kingston 16G DDR4	2
Graphic card	GEFORCE GTX1080Ti	2
Solid-state drives	Kingston 256G	1
Hard disk	Western digital 1T	1

### Model training

**Fig. 10:**
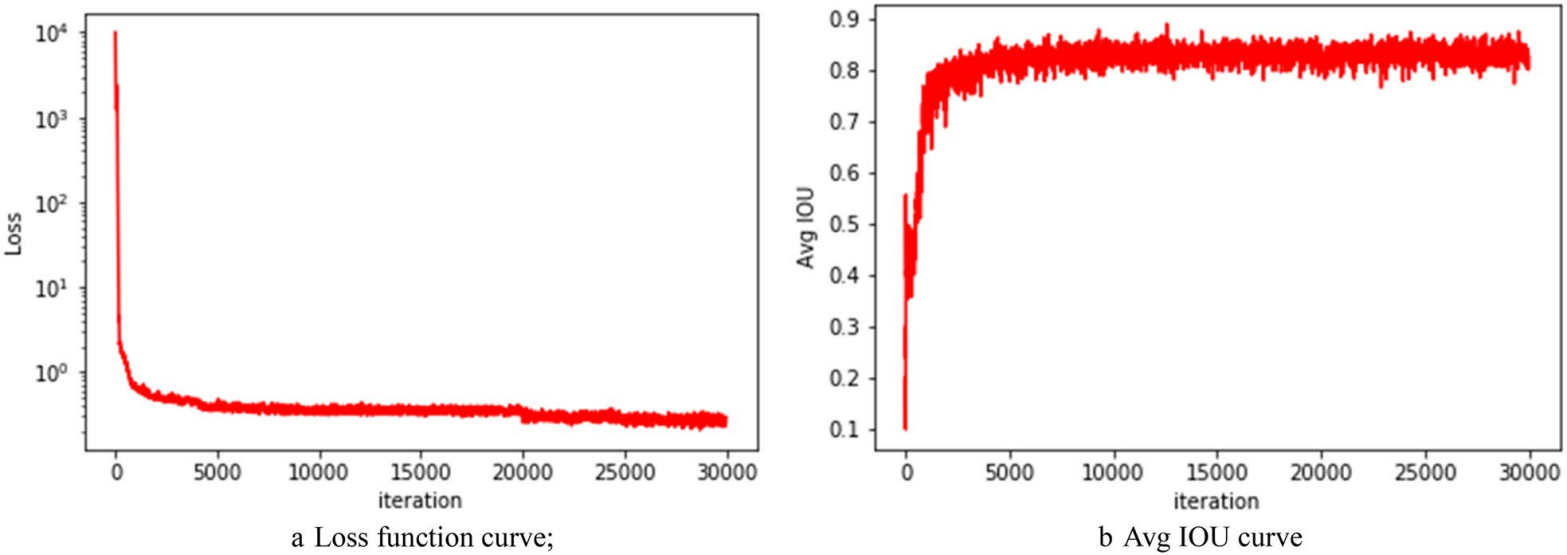
Improved YOLOv3 loss curve and Avg IOU curve

The original YOLOv3 and the improved YOLOv3 are trained separately. The initial learning rate is set to 0.001 and the attenuation coefficient is 0.0005 in the training phase. When the number of training iterations is 2000 and 25,000, the learning rate is reduced to 0.0001 and 0.00001, respectively, which further converges the loss function. The convergence curve of the loss value in the training process of the improved YOLOv3 network is shown in Fig. 10a. The Avg IOU curve of the object bounding box and ground truth is shown in Fig. 10b.

After about 30,000 iterations, the parameters tend to be stable, the final loss value drops to about 0.2, the Avg IOU gradually approaches 1, and finally stabilizes at about 0.85. From the convergence analysis of the parameters, it can be seen that the training results of the improved YOLOv3 network are ideal.

## Results and discussion

### Comparison of different detection methods

It is difficult to contrast the pre-existing literature due to different diseases of different plants in different regions and different datasets. Thus, this study presents comparative experiments to current popular algorithms. This study adopts the method of comparative experiment. It uses network model Faster-RCNN, SSD, YOLOv3 and the improved YOLOv3 to perform the comparative experiment and verify the model effect on different datasets.

Table 4 compares the test performance of this research method and various detection methods on the self-established tomato diseases and pests dataset. The F1 score and Average precision of this method are 94.77% and 91.81% respectively, which are 30.32% and 31.26% higher than the traditional HOG + SVM detection method, the reason is that the detection method can extract deep features benefiting from deep learning. Compared with Faster-RCNN, the detection accuracy of this method is also improved by 5.32%. The main reason is that this method uses anchor mechanism, FPN structure and improves the object detection accuracy of the network. The detection accuracy of this method is improved by 6.3% and 4.19% compared with SSD and YOLOv3, respectively, indicating that the backbone network of this method can maintain high resolution and open sensing receptive field, which is conducive to improving the accuracy of tomato diseases and pests detection.

**Table 4:** Comparison of different detection methods

Detection methods	F1 score (%)	Average precision (%)	Missing rate (%)	Time (ms)
HOG + SVM	64.45	60.55	31.7	7497
Faster-RCNN	89.04	86.49	14.5	4459
SSD	88.45	85.51	16.9	447
YOLOv3	91.43	87.62	7.7	54
The proposed method	94.77	91.81	2.1	55

In terms of missing detection rate, the HOG + SVM method is accurate for object location of tomato diseases and pests at normal scale, but misses detection for small-scale object detection. Faster-RCNN and SSD are not strong enough in characterizing feature maps extracted from shallow layer to cope with multi-scale detection, so they have a high rate of missing detection in multi-scale positioning. Although YOLOv3 can detect multi-scale objects, it still misses small objects with a missing detection rate of 7.7%. In this study, the algorithm extracts feature at each level and can accurately detect tomato diseases and pests objects at different scales, with a missing detection rate of only 2.1%.

In terms of detection time, compared with the traditional HOG + SVM detection method and Faster-RCNN, the method in this study has greatly improved, which is because YOLO, SSD series algorithms regard object detection as a regression problem and improve the detection speed. The detection speed of this method is generally consistent with that of YOLOv3. The main reason is that although this method uses dilated convolution (which is more time-consuming than ordinary convolution), resulting in an increase in the calculation amount of the detection network, the lightweight processing of the model is carried out. Thus, ensuring the detection speed and meeting the real-time requirements of Tomato diseases and pests detection.

### Comparison of different backgrounds of objects

The different backgrounds of objects can affect the detection accuracy of the model greatly. Therefore, different backgrounds of the objects are taken as a control variable in this study. The improved YOLOv3 model is used by the network model. Different backgrounds of test dataset verify the test results, as shown in Table 6.

**Fig. 11:**
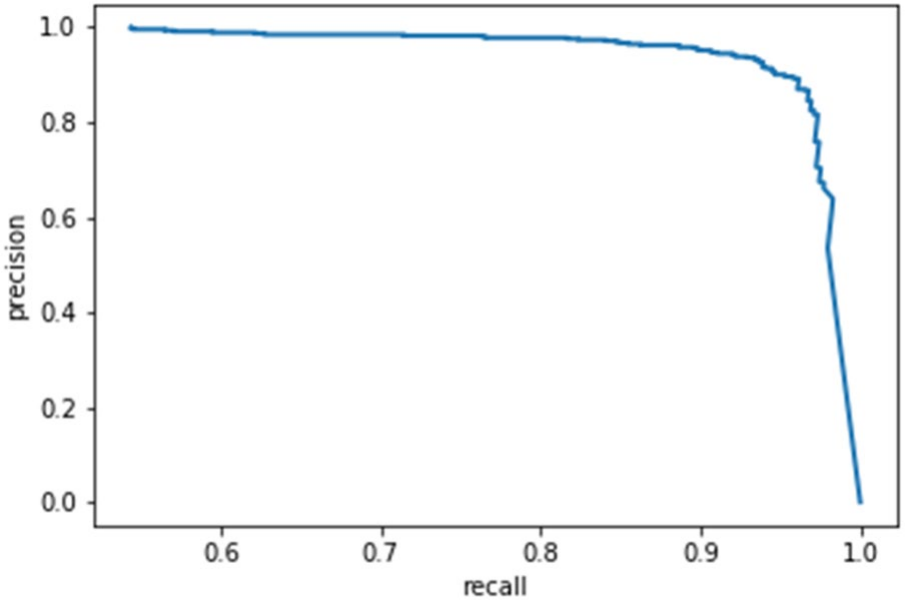
P–R curve

To recognize disease under the background of sufficient light without leaf occlusion, the F1 score of the model can reach 95.22%, the AP value can reach 92.67% and the average IoU can reach 90.89%. Table 5 shows that the detection accuracy is slightly low for recognizing disease under the background of insufficient light with leaf occlusion and shadow with leaf occlusion. The reason is that the backgrounds have elements that mimic certain disease characteristics, considering the actual application scenario. Thus, the network may learn them, which influences the recognition effect. The P–R curve of the whole test set is shown in Fig. 11.

**Table 5:** Comparison of detection results under different backgrounds

Test set	F1 score/%	Average precision/%	Average IoU/%
Sufficient light without leaf occlusion	95.22	92.67	90.89
Sufficient light with leaf occlusion	94.81	91.92	88.91
Insufficient light without leaf occlusion	94.03	90.85	86.79
Insufficient light with leaf occlusion	93.59	90.36	85.68
Shadow without leaf occlusion	92.87	90.11	85.13
Shadow with leaf occlusion	91.92	90.02	84.09

### Comparison of different class of tomato diseases and pests

The detection effect of each class of tomato diseases and pests can be analysed by Table 6.

**Table 6:** Comparison of different class of tomato diseases and pests

Class	Average precision/%			
	YOLOv3		The improved YOLOv3	
	the front side of leaves	the back side of leaves	the front side of leaves	the back side of leaves
Early blight	88.73	88.65	90.51	90.36
Late blight	89.59	89.32	90.98	90.35
Powdery mildew	91.08	92.13	92.14	92.65
Spot blight	90.66	90.02	92.05	91.68
Gray mold	90.54	90.23	91.12	90.84
Leaf mold	90.13	90.69	91.07	91.92
Gray leaf spot	91.89	91.21	93.63	93.15
Leaf miner	86.13	85.29	92.31	92.01
Whitefly	86.26	85.38	92.52	92.16

As shown in Table 6, the accuracies of early blight and late blight are relatively low, which is due to the similar textures and lesions of the leaves of the two diseases, which easily lead to misjudgement in identification. Gray leaf spot has the highest precision because the leaves of gray leaf spot had larger differences in colour and lesions than those of other diseases and pests, and the characteristics are obvious. Powdery mildew and leaf mod have no obvious characteristics in the front side of leaves in the early stage of disease onset. In contrast, the characteristics are relatively obvious in the back side of leaves. Since the dataset collected in this study is the leaves in the early stage of the diseases, the accuracy of powdery mildew and leaf mold in the back side of leaves is slightly higher than that in the front side of leaves, compared with other classes of diseases and pests. Through comparison, it is found that the improved model had better effect than the original YOLOv3 model in leaf miner and whitefly detection, and the detection accuracy in the front side of leaves is improved by 6.18% and 6.26%, respectively.

**Fig. 12:**
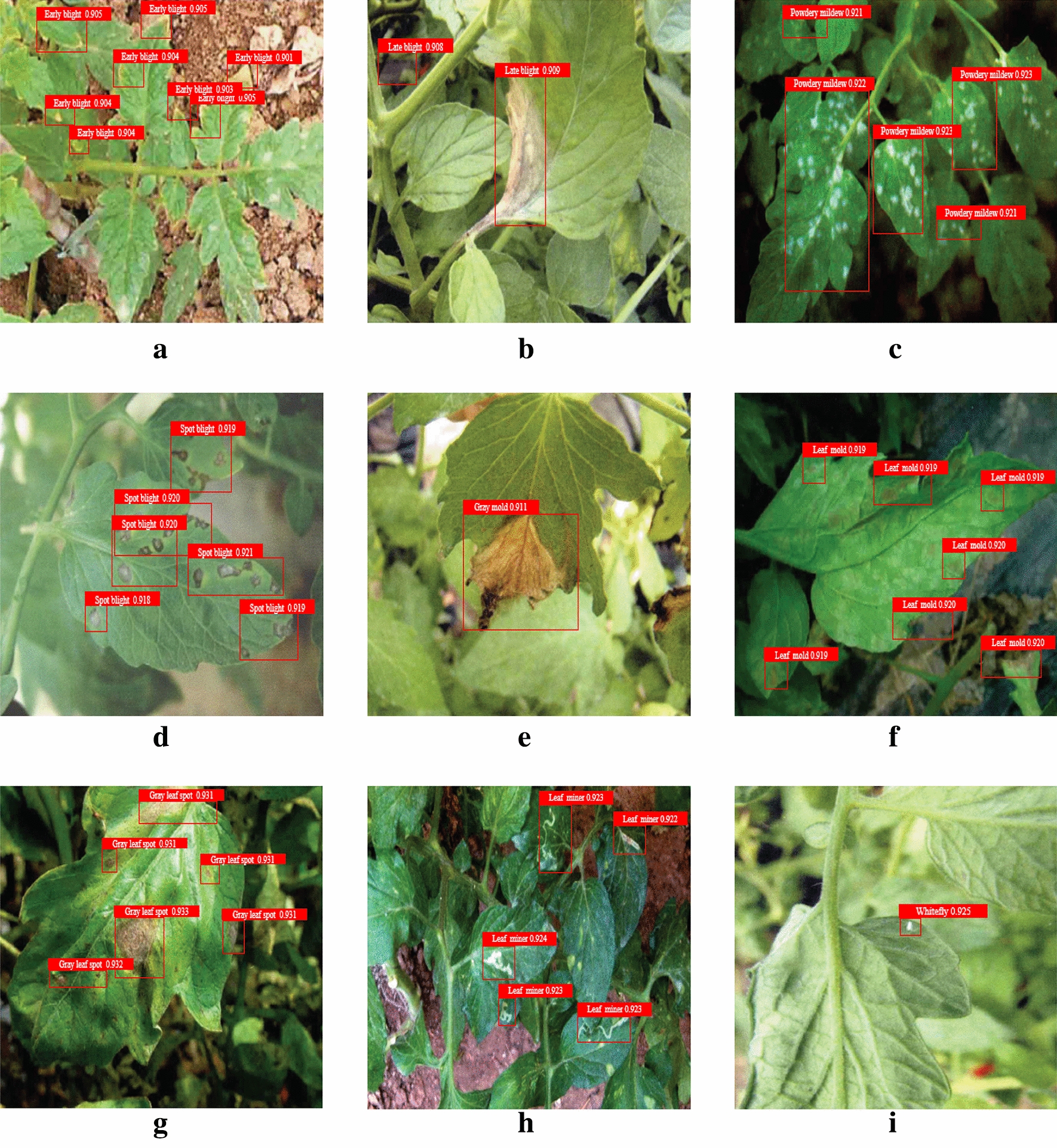
Effect diagram of the proposed detection method

Figure 12 shows the effect diagram of the proposed detection method. A, B, C, D, E, F, G, H, I are the detection results of early blight, late blight, powdery mildew, spot blight, gray mold, leaf mold, gray leaf spot, leaf miner and whitefly. Because there are a large number of invalid areas in the original image (such as soil and plastic film outside the leaves of diseases and pests), during the detection process, some of the background areas are similar to the diseases and pests in convolution characteristics, that is to say, the background areas are mistakenly regarded as diseases and pests. The proposed method can miss the detection of tomato early blight under the condition of shadow and leaf occlusion. This performance is due to the effect on the detection accuracy of tomato early blight object in a darker background.

The results of the above-mentioned comparative tests indicate that the proposed improved YOLOv3 can effectively identify tomato diseases and pests under natural environment. The recognition accuracy and speed of the proposed method have significant advantages over other methods. The high accuracy and fast detection speed make the proposed algorithm available for real-time object detection of tomato diseases and pests in complex scenarios.

### Results of the algorithm running on the unedited videos

To test the results of the algorithm running on the unedited videos, ones containing large amounts of healthy plants, several scenarios with many occurrences of tomato diseases and pests were selected to test the detection effect of the proposed model in this study, and the results are shown in Table 7. Since there is no pre-annotation in the video, this study used a manual discrimination method to conduct data statistics.

**Table 7:** Statistical result of the proposed model on the unedited videos

Unedited videos	Precision (%)	Recall (%)
Video1	89.95	81.08
Video2	89.77	81.77
Video3	90.03	82.13
Video4	90.69	82.44
Video5	90.02	81.98
Average	90.09	81.88

From Table 7, the overall precision rate of the algorithm running on the unedited videos reached 90.09% and the recall rate reached 81.88%. It can be seen that the number of misjudgements in this model is very low and the number of missed judgments is slightly higher. The experimental results show that the proposed method achieves good results on actual video data and can provide technical help and support for the intelligent management and control of tomato diseases and pests in the complex natural environment. Therefore, the proposed model can be deployed in tomato greenhouses in the future.

## Conclusions and future directions

### Conclusions

Object detection is an important research direction in the field of computer vision. The existing algorithms are difficult to achieve satisfactory results in the natural environment. To overcome the influence of the diversity of diseases and pests, changes in light and leaf occlusion on the detection accuracy, this study proposes an improved YOLOv3 detection algorithm, which improves the backbone network, NMS algorithm and loss function, thereby improving the recognition ability of diseases and pests. The results show that the average recognition accuracy of this method is 91.81%. Also, the algorithm can run successfully on the unedited videos in real natural scenario. Consequently, the proposed method can greatly improve the detection effect of small objects and leaf occlusion and can achieve good recognition effect under different background conditions, indicating that the method is feasible for the detection of tomato diseases and pests in the natural environment.

### Future directions

This study mainly studied the early detection of tomato diseases and pests under natural conditions, which can meet the accuracy and speed requirements of tomato diseases and pests detection as a whole. However, some problems still need to be solved urgently.

1. Based on tomato diseases and pests, it is necessary to extend to other kind of crops. At present, almost all crops may be affected by diseases and pests, and the loss of yield is serious. It is of great significance to identify the diseases and pests of each crop intelligently.
2. Increase the division of diseases and pests severity, study the early warning of diseases and pests. The process of diseases and pests occurrence is related to the parasitic process of bacteria, and it is a process from local leaves to the whole leaves. Although there are obvious differences in different diseases and pests, the characteristics of the same diseases and pests in different periods are also not identical. Finely distinguishing the characteristics of different development degrees of diseases and pests, and determining the severity of diseases and pests can guide managers to take corresponding remedial measures, which can not only reduce the loss of production, but also avoid excessive consumption of resources. Simultaneously, in the early stage, when plants are infected by pathogens, they will not show obvious signs immediately. It is impossible to observe whether they are infected from the appearance characteristics alone. In the future, it is necessary to use the Internet of Things sensor technology to obtain environmental information and construct a diseases and pests early warning model combined with a deep learning object detection algorithm to improve the comprehensiveness, timeliness and accuracy of diseases and pests early warning.
3. Apply the world’s leading artificial intelligence (AI) image recognition technology to the field of agricultural plant protection, collect the world’s diseases and pests collection information, coupling geographic and meteorological data, build and share large data of agricultural diseases and pests, and develop an AI-based intelligent diseases and pests identification system and integrated application project.

## Data Availability

For relevant data and codes, please contact the corresponding author of this manuscript.
